# Non food-related risk factors of campylobacteriosis in Canada: a matched case-control study

**DOI:** 10.1186/s12889-016-3679-4

**Published:** 2016-09-27

**Authors:** André Ravel, Katarina Pintar, Andrea Nesbitt, Frank Pollari

**Affiliations:** 1 Département de pathologie et microbiologie and Groupe de recherche en épidémiologie des zoonoses et santé publique, Faculté de médecine vétérinaire, Université de Montréal, St-Hyacinthe, QC Canada; 2 Center for Food-borne, Environmental and Zoonotic Infectious Diseases, Public Health Agency of Canada, Ottawa, ON Canada; 3 Center for Food-borne, Environmental and Zoonotic Infectious Diseases, Public Health Agency of Canada, Guelph, ON Canada

**Keywords:** Campylobacteriosis, Case-control, Matching, Waterborne transmission, Environmental transmission, Swimming, Raw water

## Abstract

**Background:**

Campylobacteriosis is a prominent bacterial gastrointestinal infection worldwide with several transmission pathways. Its non-foodborne routes have been less documented and quantified. The study aimed to quantitatively explore the role of potential risk factors not directly associated with food for sporadic cases of *C. jejuni* infection in Canada.

**Methods:**

This retrospective matched case-control study was built on an enhanced campylobacteriosis surveillance system and on a survey of healthy people and their behaviour with regards to potential risk factors for gastrointestinal infections that occurred in the same area in Canada. Eighty-five cases were individually matched by age and season to 170 controls.

**Results:**

Through conditional logistic regression, risk factors were found only among water-related factors (drinking untreated water, using tap filter, drinking water from well and swimming in natural water), whereas drinking bottled water was protective. Among the 32 non-water related factors explored, 12 were surprisingly ‘protective’ factors without relevant explanation for that effect (for example gardening, attending a barbecue, eating food from a fast-food restaurant), suggesting that human infection by *Campylobacter* may be more frequently acquired at home than outside the home.

**Conclusions:**

This study confirms and quantifies the importance of the waterborne transmission of campylobacteriosis. People are encouraged to drink only treated water and to avoid the ingestion of natural water as much as possible while swimming or playing in water. Globally, general hygiene and proper food handling and cooking practices at home should continue to be encouraged.

**Electronic supplementary material:**

The online version of this article (doi:10.1186/s12889-016-3679-4) contains supplementary material, which is available to authorized users.

## Background

Campylobacteriosis is the most common bacterial gastrointestinal disease reported in developed countries. Many animals serve as reservoirs, including food-production animals (poultry, cattle, swine), wildlife and pets. As a result, humans can be contaminated in various ways, and these routes are usually grouped as foodborne, contact with animals, waterborne and environmental routes [1]. The foodborne route, and chicken in particular, has been demonstrated as the most important route of transmission for human disease. The relative importance of each transmission route is still largely uncertain, as illustrated by the variability between and within studies on the proportion of human campylobacteriosis cases attributed to the foodborne pathway by expert elicitations [2–4]. Many potential risk factors have been measured and tested for their association with the disease through epidemiological case-control studies [5]. Due to differences in the definition or measure of those potential risk factors, the synthesis of numerous risk factor studies is sometimes inconclusive and do not yield to universal risk factors. For example, the meta-analysis of campylobacteriosis case-control studies performed by Domingues et al. found that both drinking water and recreational water are significant risk factors (pooled odds ratio (OR) 2.40, 95 % CI 1.76–3.26, and 1.70, 95 % CI 1.01–2.86, respectively) [5]. On the opposite according to a recent study in Spain four water-related variables were not found to be statistically associated with disease (namely tap water, bottled water, untreated water and swimming in a river or reservoir) [6]; this may have an impact on Domingues et al.’s’ pooled ORs, assuming that the quality of the Spanish study fulfils the requirement for inclusion in the meta-analysis. This illustrates that a general ‘drinking water’ exposure variable may hide more specific drinking water sources, whether it is private well water (untreated), municipal/tap water or bottled water as the main source. Even for the most important source of human *Campylobacter* infections (specifically chicken and more generally poultry), the summary of results is not always consistent across studies. For example, and according to Domingues et al.’s meta-analysis [5], eating chicken in a restaurant was a significant risk factor (pooled OR 2.06, 95 % CI 1.86–2.27), but eating chicken per se was not significant (pooled OR 1.09, 95 % CI 0.90–1.33), suggesting that the place of preparing or eating the meal has an effect on such a risk. Unfortunately, these results impede the development of a straightforward extrapolation of study findings over place and time or to more general risk factors such as drinking water.

Campylobacteriosis is also the most prominent bacterial gastrointestinal infection in Canada with an estimated total incidence of 213,749 domestically-acquired cases (90 % credible interval 144,288–308,837) per year [7]. To date, few epidemiological case-control studies have been conducted to identify specific risk factors for sporadic human campylobacteriosis in Canada. A matched case-control study of sporadic cases of campylobacteriosis in one small region in the province of Quebec in 2000–2001, mostly focussed on risk factors related to food, outlined the handling and/or consumption of several food products (e.g. chicken, turkey, raw milk) as well as contact with animals through occupational activities or visiting a farm or a zoo [8]. However, the study results did not explain the seasonal pattern and it was hypothesized that environmental exposure, such as water, may drive the observed seasonality in human cases. The authors later compared cases from rural areas to cases from urban areas in the same region and found that working with animals and using well as source of household water were significant risk factors [9]. A recent case-case study, using other infectious enteric disease cases as controls, highlighted private well water as risk factors for sporadic campylobacteriosis in British Columbia, Canada [10]. Beyond foodborne exposure, this limited number of Canadian case-control studies indicate that contact with animals and water are two important exposures to *Campylobacter*, and that better quantification is necessary for attributing human campylobacteriosis to source. Although Canada has one of the safest drinking water supplies in the world, sources of drinking water can still become contaminated through agricultural run-off, wastewater treatment effluent and wildlife [11].

Exploring, confirming and precisely quantifying risk and protective factors of human campylobacteriosis is complicated by several microbiological and epidemiological features of the disease. First, the *Campylobacter* genus encompasses several species that are pathogenic to humans, primarily *C. jejuni* and *C. coli*, with each having specific bacteriological, biological or epidemiological characteristics leading to some different risk factors [12]. Second, a seasonal pattern in human campylobacteriosis has been repeatedly observed in various temperate parts of the world, but no definitive explanation has been provided [13–15]. Some environmental features relevant to the presence and transmission of *Campylobacter* (e.g. prevalence and concentration of *Campylobacter* in water bodies) may well follow a seasonal pattern [16]. Some potential risk factors can also be seasonal, such as outdoor activities, visiting farm animals, attending a barbeque, especially in countries like Canada where the seasons are well marked [17]. Third, age-specific risks have also been documented especially among the young (<15 years), the late teens to early 20s (18–24 years) or the older (>60 year) population [15, 17–21]. Fourth, recent epidemiological studies have reported that exposures in rural areas versus those in urban areas may not be the same [9, 17, 18, 22, 23]. Finally, human campylobacteriosis is most often sporadic and outbreaks are rare. In addition, up to 20–30 % of all reported cases are travel-acquired [24]. Several case-control studies have been undertaken worldwide and were recently reviewed by Domingues et al. [5], which summarized the association between several potential risk factors and the disease. While of great interest in providing an overview of general potential risk factors, it provides limited answer to more specific risks associated with specific *Campylobacter* species, age and gender, season and place of residence (urban vs. rural).

The aim of this study was to quantitatively explore the role of potential risk factors not directly associated with food for sporadic cases of *C. jejuni* infection, with consideration of season and age.

## Methods

The study is a retrospective matched case-control study using data from an enhanced campylobacteriosis surveillance system and from a survey of healthy people and their behaviour with regards to potential risk factors for gastrointestinal infections that occurred in the same area.

### Study population

The study population was the Region of Waterloo (ROW), Ontario, Canada (http://gis.region.waterloo.on.ca). It is comprised of three cities and four townships, with a population of approximately 527 thousand people in 2010 (Population and Household Estimates, Regional Municipality of Waterloo, accessed October 2011) and roughly 70 % urban and 30 % rural. The study period spanned the time period from August 2009 to July 2010, inclusive.

### Data sources and subject selection

Campylobacteriosis is a reportable disease in Canada. In addition to the existing laboratory-based surveillance system in Canada, campylobacteriosis cases reported to ROW were systematically interviewed by public health inspectors using an enhanced questionnaire since mid-2005 when this area became the first sentinel site of the National Integrated Enteric Pathogen Surveillance System (FoodNet Canada). The detailed information collected through the standardized follow-up questionnaire addresses demographic characteristics and disease symptoms, as well as exposures to potential risk factors that may have occurred within the 10 days prior to the case’s disease onset (see Additional file 1). Outbreak-related cases were identified by ROW Public Health on the basis of epidemiological or laboratory evidence. Travel-related cases were defined as cases that travelled outside Canada prior to the disease onset and for which the exposure had likely occurred abroad considering the travel dates and the incubation period [24]. Ethics approval for the surveillance data collection was obtained through the Region of Waterloo Public Health Ethics Review Committee in 2005.

The controls were selected from a dataset generated through a study that involved a randomized, population-based telephone survey of healthy residents in the same area [25]. The survey was administered over a 12-month period (August 2009 to July 2010), with 1200 respondents interviewed, uniformly spread over the year and between three different recall periods (i.e., 3, 7 and 14 days). The survey questionnaire was similar to the one used for the systematic follow-up of campylobacteriosis by ROW public health inspectors. Individuals were randomly assigned to one of three recall periods (3 day, 7 day or 14 day). Individuals with diarrhoea or vomiting in the recall period prior to the interview were excluded. Interviews of children under 18 years old required parental or legal guardian consent; the respondent could be a proxy or the child itself. The survey was approved by the Public Health Agency of Canada Research Ethics Board (REB-2008–0040) and the University of Waterloo Office of Research Ethics (ORE# 15764). Further details are provided in David et al. 2013 [25]. For the present work, two controls were manually selected for each case with individual matching on age and time of the year: the triplet (i.e. case matched to two controls) must be within the same age group (0–4, 5–14, 15–29, 30–44, 45–59, 60+ years old); and the date of the control’s interview must be within 14 days of the case’s onset date. Where more than two controls were possible, the controls with the interview date the closest to the case’s onset date were chosen. When onset date was missing, it was estimated by subtracting the median time between onset and report dates calculated for those cases with both dates available (8 days). Because the incubation period of campylobacteriosis is typically 3–5 days with a range of 1–10 days, only controls assigned to the 7 or 14 day recall periods were eligible.

### Variable measurement

Questions common to both the case and the control questionnaires included demographics (age, gender and occupation) and exposure to potential sources of enteric pathogens, such as water (drinking and recreational water), environment (country property, animals, gardening, outdoor activities), travel (within Canada), food supply (type of shop for usual food and meat purchases, origin of the food coming from outside the home), high-risk foods (unpasteurized products (dairy, juice, milk), spoiled and undercooked food) and social events (barbequing, social gathering).

Some variables were re-categorized prior to analysis, either by categorizing text questions, or by aggregating questions related to the same category of exposure. Occupation was categorized into agriculture/food processing, food preparation, daycare service, healthcare service and another category regrouping any other occupation (and considered not at specified high-risk with regards to campylobacteriosis). The animals that people were exposed to when living on or visiting a farm, country property or petting zoo were extracted from the text question and categorized into cattle, cat, dog, rodent, rabbit, poultry, other poultry (i.e. not chicken), horse, sheep, reptile, pig, llama and other. Some variables were merged for the analyses: all recreational water exposures other than “pool”, the consumption of all unpasteurized products, exposures to all food animals when living on or visiting a farm, country property or petting zoo, and contact with all pets other than cats and dogs. Finally, the potential risk factors considered were partitioned into exposure groups: waterborne route of exposure, environmental exposure, social event and domestic travel, food purchasing behaviour, food outside the home, high-risk food and high risk occupation (Table 1).

**Table 1:** Description of cases and controls and univariate analysis results

	Cases		Controls		Wald chi-square		
	*n/N*	%	*n/N*	%	*p*-value	mOR^a^	95 % Wald CI
Waterborne routes of exposure							
Drinking water source = private well	12/85	14	12/170	7	0.08	2.1	0.91–4.7
Drinking water source = municipal water	55/85	65	125/170	74	0.15	0.67	0.38–1.2
Drinking water source = bottled water	38/85	45	99/170	58	0.042	**0.58**	0.34–0.98
Use any in home treatment	33/81	41	78/170	46	0.44	0.81	0.47–1.4
Use tap filter	15/85	18	11/170	6	0.007	**3.1**	1.3–7.1
Drink untreated or raw water	11/76	14	6/169	4	0.004	**4.6**	1.6–13.0
Swim in or go into any natural water	11/85	13	11/170	6	0.082	2.3	0.90–5.8
Swim or go into any artificial water	14/85	16	48/170	28	0.027	**0.43**	0.20–0.91
Environmental exposures							
Go canoeing, kayaking, hiking or camping	6/84	7	15/170	9	0.64	0.79	0.30–2.1
Live on a farm or country property	10/84	12	9/170	5	0.068	2.5	0.94–6.7
Live with contact with food animals	6/85	7	5/170	3	0.15	2.4	0.73–7.9
Visit a farm or country property	14/85	16	24/170	14	0.60	1.2	0.58–2.6
Visit farm or country property and contact with food animals	8/85	9	18/170	11	0.75	0.86	0.33–2.2
Contact with households pets	52/84	62	121/170	71	0.12	0.64	0.36–1.1
Contact with cat	19/85	22	67/170	39	0.008	**0.45**	0.25–0.82
Contact with dog	44/85	52	89/169	53	0.89	0.97	0.58–1.6
Contact with pets other than cats and dogs	4/85	5	24/170	14	0.025	**0.28**	0.09–0.85
Gardening	12/83	14	59/170	35	0.0007	**0.29**	0.14–0.59
Social events and domestic travel							
Attend a barbeque	25/82	30	84/170	49	0.005	**0.42**	0.23–0.77
Attend social gathering	17/83	20	63/170	37	0.016	**0.46**	0.25–0.87
Travel outside the region but within Canada	8/85	9	34/170	20	0.031	**0.39**	0.16–0.92
Food purchasing behaviours							
Shop for food in supermarket	78/81	96	165/170	97	0.79	0.81	0.17–3.8
Shop for food in farmers market	7/81	9	51/170	30	0.0005	**0.23**	0.10–0.52
Shop for food in butcher shop	6/81	7	34/170	20	0.016	**0.33**	0.13–0.81
Shop for food in farm laneway, farm stand	3/81	4	14/170	8	0.16	0.39	0.11–1.4
Shop for food from other location	4/81	5	6/170	4	0.74	1.2	0.35–4.4
Meat purchasing behaviours							
Eat meat purchased from another place than grocery store	13/85	15	43/169	25	0.084	0.55	0.27–1.1
Consume meat from hunting	1/85	1	6/168	4	0.31	0.33	0.04–2.8
Consume meat from butcher	7/85	8	31/169	18	0.042	**0.41**	0.17–0.97
Consume meat from kill	4/85	5	11/169	7	0.57	0.71	0.22–2.3
Food outside home							
Eat food prepared outside the home	45/76	59	135/169	80	0.001	**0.36**	0.19–0.67
Food from fast food chain restaurant	19/85	22	86/168	51	<0.0001	**0.26**	0.14–0.50
Food from eat-in restaurant	26/85	31	88/169	52	0.002	**0.41**	0.23–0.72
Food from eat-in cafeteria	2/85	2	14/169	8	0.087	0.27	0.06–1.2
Food from deli	1/85	1	14/160	9	0.052	0.13	0.02–1.0
Food from ready-to-eat	5/85	6	55/169	33	<0.0001	**0.12**	0.04–0.34
Food from food vendor	3/85	4	17/169	10	0.08	0.32	0.09–1.1
High risk foods							
Consume undercooked food	8/85	9	24/169	14	0.30	0.64	0.28–1.5
Consume spoiled food	9/84	11	14/169	8	0.54	1.3	0.55–3.2
Consume unpasteurized products (dairy, juice, milk)	5/84	6	18/169	11	0.28	0.58	0.21–1.6
High risk occupations							
Agriculture/food and animal processing	2/85	2	3/170	2	0.75	1.3	0.22–8.0
Food preparation	4/85	5	3/170	2	0.17	3.3	0.59–19
Daycare	2/85	2	4/170	2	Not converged		
Healthcare	3/85	4	5/170	3	0.80	1.2	0.29–5.0
Gender							
Male	45/85	53	72/170	42	0.08	1.7	0.94–3.1

^a^Matched odd ratio, bold indicates significant association (*p* < 0.05)

### Analysis

Matched univariate analysis was conducted on all variables to estimate crude matched odds ratios (mOR) and their 95 % confidence intervals. Conditional logistic regression was undertaken separately on each exposure group using a manual sequential backward procedure with a *p*-value > 0.15 to remove the variable. Potential confounders were considered whenever the point estimates of the variable coefficients in a model changed >20 % with the potential confounder present.

To build the final multivariate model, conditional logistic regression was conducted starting with all variables that had been retained in each exposure group model. A manual backward procedure was applied with a *p*-value of 0.05. Once the final, parsimonious multivariable model was developed, biologically plausible interactions between the main effects were tested. Significance in all modelling was assessed based on Wald’s statistics. Adjusted matched odds ratio (amOR) and their 95 % confidence interval were computed for each variable remaining in the final model. The model fit was assessed according to Hosmer and Lemeshow [26]. The Pearson residuals (r), the leverages (h), the standardized Pearson residuals and its squared value measuring the lack of fit (ΔX^2^), and the influence diagnostic delta beta (Δb) were computed and plot against the predicted values. Observations with divergent values, i.e. −3 ≤ r ≥ 3, h > 0.3, ΔX^2^ > 8, and Δb > 1, were considered outliers. Running and comparing models with and without outlying triplets was performed to assess their influence on the final model. Missing values and ‘Unsure’ responses were omitted when computing any statistics. All descriptive and logistic regression analyses were run on SAS (Version number 9.1, SAS Institute Inc., Cary, NC, USA).

The proportion of all sporadic, domestically-acquired cases of *C. jejuni* infection that would be prevented by removing one of the observed risk factors, assuming its causal and its effect measured accurately, namely its population attributable fraction (PAF) was computed for all risk factors based on their amOR according to the following formula (Eq. 1):

$$ \mathrm{P}\mathrm{A}\mathrm{F}={\mathrm{p}}_{\mathrm{e}}\times \left[\left(\mathrm{amOR}{\textstyle \hbox{-} }1\right)/\mathrm{amOR}\right] $$

where p_e_ is the proportion of cases exposed to the risk factor [27]_._ The PAF 95 % confidence intervals were estimated according to the package punafcc for Stata and run on Stata/IC (Version number 13.1, Stata Corp, College Station, TX, USA).

In order to discuss the study findings, the value of the odds ratio the study was able to statistically detect at the univariate analysis step was estimated according to the method proposed by Dupont [28]. This method allows for estimating the minimum odds ratio a matched case control study would detect according to the number of cases, the number of controls matched per case, the values set for the type I error and the power, the correlation between cases and controls with regards to the exposure, and various values for the probability of the controls of being exposed. For this estimation, the type I error probability was set at 0.05, power at 0.80 and the correlation between cases and controls with regards to exposure at 0.2, a complete independence (null correlation) being not real. The number of cases used for this estimate was the number of cases available during the study period that could be matched with two controls.

## Results

**Fig. 1:**
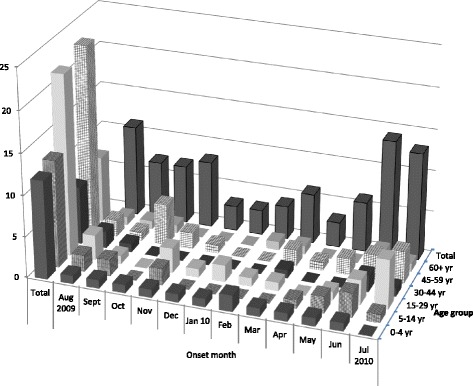
Distribution of the 85 cases by age group and month of onset

Over the study time period, 98 cases of *C. jejuni* non outbreak-related, domestically-acquired gastrointestinal infection were reported to and interviewed by ROW Public Health, and 775 controls were available for matching: 375 controls had been interviewed for the 7 day recall period and 380 for the 14 day recall period. Two controls were individually matched on age group and dates for 85 cases (87 % of the available cases). The distribution of cases by age group and month of disease onset is shown in Fig. 1 and is similar in relative numbers for the controls. Few cases (and controls) were among the 60+ year age group (*n* = 8 cases) and the 30–44 year age group (*n* = 7 cases). Forty-seven percent of cases were female, versus 58 % of controls (Fisher’s exact test *p*-value = 0.11). Overall, 38 (45 %) cases occurred during the summer months (August 2009, and June and July 2010). The 170 selected controls were equally divided between the two recall periods (86 among the 7 day recall period vs. 84 among the 14 day recall period). This uniform distribution of controls by recall period was true when age group or month were considered (Fisher’s exact test *p*-value = 0.90 and 0.98, respectively).

Table 1 shows the distribution of the independent variables among the cases and the controls with the results of the univariate testing. Seventeen variables were significantly associated with the probability of being a case, at the 0.05 level of significance. Two potential risk factors (mOR > 1) were related to water: ‘Drink untreated or raw water’ (mOR = 4.6, 95 % CI 1.6–13.0, *p* = 0.004), and ‘Use of tap filter’ (mOR = 3.1, 95 % CI 1.3–7.1, *p* = 0.007). The other 15 statistically significant variables had a mOR below 1 and were categorized into four exposure groups: ‘Environmental exposures’, ‘Social events and traveling’, ‘Food purchasing behaviour’ and ‘Food outside home’. In addition to the 17 variables significant at 0.05, 11 other variables were significant at a *p*-value between 0.05 and 0.15, and thus considered in the multivariate models. Five of them had a mOR above 1: ‘Drinking water source = private well’ (mOR = 2.1, 95 % CI 0.91–4.7, *p* = 0.08), ‘Swim in or go into any natural water’ (mOR = 2.3, 95 % CI 0.90–5.8, *p* = 0.082), ‘Live on a farm or country property’ (mOR = 2.5, 95 % CI 0.94–6.7, *p* = 0.068), ‘Live with contact with food animals’ (mOR = 2.4, 95 % CI 0.73–7.8, *p* = 0.15), and being a male (mOR = 1.7, 95 % CI 0.84–3.1, *p* = 0.08). No variables among the ‘High risk food’ and the ‘High risk occupation’ groups had a *p*-value ≤0.15. The univariate analysis with our design of 85 cases and two matched controls per case was able to detect statistically significant associations between disease and exposure with mORs below 0.33–0.4 or above 2.5–3.0 with a power equal to or greater than 0.8 and an alpha value of 0.05, when the probability of exposure in controls was between 0.20 and 0.70.

The final models by exposure group are presented in Table 2. For the waterborne routes of exposure, three variables remained in the model, two as potential risk factors (‘Use tap filer’: amOR = 5.1, 95 % CI 1.9–13, *p* = 0.01; ‘Drink untreated or raw water: amOR = 3.3, 95 % CI 1.1–10, *p* = 0.037) and one as potential protective factor (‘Swim in or go into artificial water’: amOR = 0.35, 95 % CI 0.14–0.86, *p* = 0.023).

**Table 2:** Multivariate analysis results within each exposure group

Potential risk factors by exposure group	Beta coefficient	S.E.	Wald statistic	*P* value	maOR^a^	95 % CI	
Waterborne routes of exposure							
Drinking water source = private well	R^b^						
Drinking water source = municipal water	R						
Drinking water source = bottled water	R						
Use tap filter	0.816	0.250	10.7	0.001	**5.1**	1.9	13
Drink untreated or raw water	0.593	0.285	4.3	0.037	**3.3**	1.1	10
Swim in or go into any natural water	R						
Swim or go into any artificial water	−0.526	0.231	5.2	0.023	**0.35**	0.14	0.86
Environmental exposures							
Live on a farm or country property	R						
Live with contact with food animals	R						
Contact with households pets	R						
Contact with cat	−0.365	0.158	5.4	0.0205	**0.48**	0.26	0.89
Contact with pets other than cats and dogs	R						
Gardening	−0.574	0.190	9.1	0.0025	**0.32**	0.15	0.67
Social events and domestic travel							
Attend a barbeque	−0.352	0.157	5.0	0.025	**0.50**	0.27	0.92
Attend social gathering	−0.358	0.168	4.5	0.033	**0.49**	0.25	0.95
Travel outside the region but within Canada	R						
Food purchasing behaviours							
Shop for food in farmers market	−0.742	0.213	12	0.0005	**0.23**	0.10	0.52
Shop for food in butcher shop	R						
Eat meat purchased from another place than grocery store	R						
Consume meat from butcher	R						
Food outside home							
Food from fast food chain restaurant	−0.527	0.175	9.1	0.0025	**0.35**	0.18	0.69
Food from eat-in restaurant	−0.353	0.166	4.5	0.033	**0.49**	0.26	0.95
Food from eat-in cafeteria	R						
Food from deli	R						
Food from ready-to-eat	−0.862	0.289	9.2	0.0024	**0.18**	0.059	0.54
Food from food vendor	R						

^a^Matched adjusted odd ratio (bold indicates statistically significant results)

^b^R = removed during the backward regression process

The final multivariate model, initially including gender and the significant variables developed for each exposure group model, contained six statistically significant variables (Table 3). Gender was not significant. Two of the six final variables were water-related risk factors: ‘Use tap filter’ (amOR = 7.5, 95 % CI: 1.9–29, *p* = 0.004) and ‘Drink untreated or raw water’ (amOR = 8.1, 95 % CI 1.7–38, *p* = 0.008). The interaction between these two variables was tested but was not significant (Wald chi-square = 0.0001; p-value = 0.99). Six cases and three controls showed poor fit based on their ΔX^2^ (ΔX^2^ between 6.3 and 12). Of these 9 subjects, one case and one control from the same triplet showed greater Δb values compared to the other subjects (Δb ≈ 0.93). The multivariate model run without this triplet did yield similar results and no outlying subjects. We concluded that the first model based on all triplets showed a good fit.

**Table 3:** Final multivariate analysis results

Potential risk factors	Beta coefficient	S.E.	Wald statistic	*P* value	maOR﻿^a﻿^	95 % CI	
Use tap filter	1.00	0.349	8.27	0.004	**7.5**	1.9	29
Drink untreated or raw water	1.05	0.395	7.02	0.008	**8.1**	1.7	38
Swim or go into artificial water	R^b^						
Gardening	−0.751	0.270	7.76	0.005	**0.22**	0.08	0.64
Contact with cat	R						
Attend a barbeque	−0.654	0.237	7.64	0.006	**0.27**	0.11	0.68
Attend a social gathering	R						
Shop for food in farmers market	R						
Food from fast food chain restaurant	−0.559	0.236	5.61	0.018	**0.33**	0.13	0.82
Food from eat-in restaurant	R						
Food from ready-to-eat	−1.06	0.360	8.76	0.003	**0.12**	0.03	0.49
Male	R						

^a^Matched adjusted odd ratio (bold indicates statistically significant results)

^b^R = removed during the backward elimination process

The population attributable fraction was 15.1 % (95 % CI 4.8–24.4 %) for ‘Using tap filter’ and 13.3 % (95 % CI 4.1–21.7 %) for ‘Drinking untreated or raw water’.

## Discussion

This case-control study was undertaken to increase our knowledge and understanding of several potential risk factors for campylobacteriosis not directly related to food consumption. Environmental transmission, including waterborne transmission and the transmission via contact with animals, as well as food purchasing habits (type of food establishment) were the focus of this analysis since they have been less extensively studied compared to specific food risks (e.g. chicken). The analyses focussed on *Campylobacter jejuni* infection as most cases are *C. jejuni* and other *Campylobacter* species- or subtypes have some specific risk factors [12, 18, 29, 30]. The matched design allowed for control of age and season, two factors known to be confounders of the relationship between exposure and the disease [15, 17, 18, 20, 21]. The analysis was based on data collected from questionnaires for campylobacteriosis cases reported through an enhanced surveillance system and on data from a survey of healthy individuals from the same defined geographic region. The study addresses some knowledge gaps within Canada, where only two case-controls studies have previously been published on campylobacteriosis [8–10].

The study results illustrate the importance of the waterborne route of transmission: five of the eight variables used to measure various ingestion of, contact with or protection from potentially contaminated water were significantly associated with the outcome. Two variables, namely ‘Drink untreated or raw water’ and ‘Use tap filter’ were the only two risk factors in the final multivariate model. This further supports the importance of the waterborne transmission route found through a substantial review on the issue [1], and documented through outbreaks [31–36], and epidemiological analyses of sporadic cases with or without consideration of molecular speciation data [5, 9, 21, 29, 37, 38]. The proportion of campylobacteriosis cases attributed to water-related factors in our study has not comparable figures in the literature; nonetheless it was within the same range of magnitude as reported through other approaches. According expert elicitation, 10 % of campylobacteriosis cases were attributed to water exposure in Canada [39] whereas 21 % of the cases were attributed to environmental transmission, including contaminated water (drinking water, recreational water), soil, air or other environmental media in The Netherlands [3], and 1.4 % (95 % CI: 0.4–3.2 %) of human cases were attributed to drinking water and 1.9 % (95 % CI: 0 . 8–3.9 %) to recreational water in the Canterbury region of New Zealand [3]. The PAF value depends on the OR value and on the proportion of the population exposed. For the two water-related risk factors, the amOR values in the final multivariate model were greater than their respective value in the univariate model and in the model for waterborne exposure group. This greater value certainly leads to a higher proportion of cases that could be attributable to water.

The water-related variables that were statistically associated with *Campylobacter* infection in this study were biologically and epidemiologically coherent with the current understanding of this disease. Raw, untreated water consumption as a risk factor has previously been reported [31, 40]. There is ample evidence that *Campylobacter* is present in a wide range of untreated surface water, including in the FoodNet Canada sentinel site watershed(s) in Canada. Swimming is also a well-known risk factor [21, 29, 37, 41, 42]. Our study distinguished between natural recreational water and artificial water (including pool and hot tub) exposure. Swimming in natural water was found to be a risk factor, whereas swimming in artificial waters was not, likely because chlorine, commonly used as a disinfectant in pools/hot tubs/splash pads is an effective disinfectant for *Campylobacter* and an effective public health intervention for reducing risk [43].

In our study, private well as the main drinking water source was a borderline significant risk factor (at the 0.1 level) by univariate analysis (mOR = 2.1; 95 % CI 0.91–4.7) as found by others, including in Canada [9, 10, 30, 40, 42, 44]. Private well water is not always treated, and thus contributes to the overall burden of waterborne illness in Canada [11]. The bottled water variable is more difficult to interpret. Bottled water as the main source of drinking water was found to be a protective factor at the univariate analysis stage (mOR = 0.58; 95 % CI 0.34–0.98). If bottled water is treated to meet the national drinking water guidelines [45], drinking bottled water could be considered protective, compared to drinking untreated water, whether from a well or other source. This protective effect was documented previously in one campylobacteriosis waterborne outbreak [46]. However, globally, the literature shows that drinking bottled water is associated with an increased risk of campylobacteriosis, especially for travel related cases or *C. coli* infection compared to *C. jejuni* infection [12, 47–50], drinking bottled water in such cases could be a proxy for high risk exposure during travelling [48]. With regard to sporadic cases, one study found that bottled water consumption was a strong risk factor for campylobacteriosis (final adjusted OR = 1.41, 95 % CI 1.02–1.95, *p* = 0.04) with a PAF of 12 % (95 % CI 0–23) in Cardiff, UK [50]. Microbial and chemical risks associated with bottled water consumption are attributed to contaminated source water, ineffective disinfection/treatment or contamination during bottling, in Canada and internationally [51].

The interpretation of the importance of the reported ‘Use tap filter’ as a risk factor for campylobacteriosis is less straightforward. This variable was included in the follow-up questionnaires for cases and healthy controls in this study to initially understand the efficacy of home treatment in the reduction of disease. Home water treatment (carbon filters in particular) are marketed to consumers as a means to reduce both chemical and microbial exposure risks from tap water. However, in this study, the use of a tap water filter is associated with an increased risk of campylobacteriosis. There is no evidence in the literature that would suggest *Campylobacter* grows in these tap filters. We did not ask specific question about the purpose, use and maintenance of the tap filters. We propose that this variable is potentially a proxy for some other risk in the home or behaviour. Future studies will help to determine the significance of this finding.

Beyond the water-related risk factors, no other statistically significant risk factors were identified in the data. However, one third (12 out of 32) of the variables among the ‘Environmental exposures’ , the ‘Social events and traveling’ , the ‘Food purchasing behaviours’ , the ‘Meat purchasing behaviours’ and the ‘Food outside home’ groups were protective, with matched odds ratio significantly below 1. These variables were chosen for two reasons. The first reason is that they have been demonstrated as true risk factors in other studies (see Domingues and collaborators [5]) and these include contact with pets, attending a barbeque, attending a social gathering, eating food at a restaurant for example. The second reason is because the variables could increase the exposure to *Campylobacter*, like gardening which represents contact with the environment and soil, which could be contaminated by manure or directly by animals, like wild birds or outdoor pets [38]. There is no rationale to consider all those variables as truly protective. Rather, we believe that this finding suggests that overall, cases were more likely to stay at home or be less active (e.g. no gardening, no barbeque, no social gathering, no traveling, no food outside home) and not in contact with animals (e.g. little contact with pets other than dogs). There are two main hypotheses to explain this finding that cases were more likely to stay at home and were not exposed to what we tested. The first hypothesis is that either the case orthe control groups were not representative of their respective populations or there was a differential bias of information between the two groups. The cases available and used for the study might have been different from typical *Campylobacter* cases. Nevertheless they were lab-confirmed cases and reported exposures at levels similar to those observed when all reported cases were considered (data not shown). The controls may not well represent the general population at risk of getting campylobacteriosis with regards to their exposures. This may be possible since the overall healthy control study included more women than men [25], that behaviour of men and women are generally not the same with regards to risks and exposures, and our matching did not consider gender (although gender was not significant nor a confounder in our final model). The recall period was 14 days for half of the controls, which is longer by a few days than the recall period for the campylobacteriosis cases, meaning that half of the controls had a greater probability to report some exposures because of their longer recall period. Bias is possible due to the fact that the controls were not a perfect representative of the population and the greatest validity of any study result cannot be claimed. The questions asked to cases and to controls were similar or very closed. The context and method to collect were different however: the cases were identified through a surveillance system with inherent delay between the disease onset and the time of the interview by public health inspectors about what occurred prior to disease onset whereas the controls were phoned and asked to answer the questions for their activities over the last days. We can hypothesize that controls were less prone to forget their activities than the cases, implying a possible differential information bias. However such bais would hold for episodic activities (e.g. eating out) but not for permanent potential exposure (e.g. having a pet). Consequently, we do not believe such sampling bias or information bias would totally explain all those apparently protective factors. The second main hypothesis is that a large proportion of campylobacteriosis cases actually are due the contamination at home while handling, preparation or consumption of contaminated food. This is supported by the evidence that the food, and especially chicken meat, is the predominant route of transmission. The fact that campylobacteriosis cases are mostly sporadic and rarely outbreak- or cluster-related supports an exposure that is limited to one or a few people at a time, which is more likely to occur at home compared to restaurants, community meals or meals served in institution. Several outbreaks revealed that a relatively large proportion of outbreaks are linked to the private home: 7 out of 11 outbreaks investigated in Canada during 2000–2004 [52], 33 out of 225 foodborne outbreaks that occurred in the USA during 1998–2007 [53], around 50 % of the outbreaks reported in Europe in 2004–2005 [54], and 37 % of 211 outbreaks investigated in New Zealand [55]; clearly indicating that acquiring *Campylobacter* infection at home is not uncommon. The finding of unexpected ‘protective’ factors in this study is aligned with the hypothesis that contamination occurs often at home compared to other settings.

Our study was limited by the number of sporadic, domestically-acquired cases of campylobacteriosis reported during the 12-month time frame and by their matching with the controls. Ninety-eight cases were available, but 13 could not be matched resulting in 85 usable cases, leading to a limited statistical power to detect risk factors with an OR below 2.5. Using all available cases and all available controls may have increased statistical power, but we preferred to control for age and season to get more valid estimates. Obviously, because of matching, the study design did not allow to derive age- or season-specific ORs. Place of residence (rural vs. urban) and socio-economic status are other determinants of campylobacteriosis [9, 17, 18, 56, 57]. They should ideally be controlled for, but their matching would not be possible in addition to that for age and season. As a result, the risk factors highlighted in this study are not controlled for socio-economic status, a major determinant of the disease [56, 57]. The study purposely focuses on non-food-related risk factors (i.e. any specific food commodity or precise food item), thus it did not confirm or refute any food previously found associated with the disease. In addition, it did not consider other risk factors known for campylobacteriosis, like the intake of proton-inhibitor drug or suffering from other health problems [18, 58–61]. Actually, the investigated risk factors were considered unrelated to the main food-related and medication-related known risk factors for campylobacteriosis in the lack of evidence for such association. The transmission of *Campylobacter* from its reservoirs to human beings is highly complicated and numerous factors can have an influence on this transmission or the manifestation of the disease. Future work should be to undertake a comprehensive case-control study that would consider all transmission pathways and risk factors in the Canadian context as it has been done nationwide in other countries [62, 63]. Alternately, more targeted studies like our study and previous ones provide a partial picture of the risk factors. Results from one study should be interpreted with the restriction its design imposes and in the light of the findings of other studies. This study affirms the importance of the waterborne transmission in the Canadian context.

## Conclusions

This study provides additional evidence to inform our understanding of the public health risks of waterborne transmission of *Campylobacter* in Canada, with drinking raw, untreated water and swimming in natural water (rivers, lakes, streams) being confirmed as risk factors. The use of tap filter may be a risk factor for *Campylobacter*. Future studies should be undertaken to better characterize the main source of drinking water and any at-home or individual water treatment and their association with campylobacteriosis. People are encouraged to drink only treated water and to avoid the ingestion of natural water while swimming or playing in surface water. In addition, this study illustrates that human infection by *Campylobacter* may be acquired more frequently at home than outside the home. General hygiene and proper food handling and cooking practices at home should continue to be promoted in consumer education messages.

## Additional file

Additional file 1: Campylobacter Worksheet. (PDF 45 kb)

## References

[1] Whiley H, van den Akker B, Giglio S, Bentham R (2013). The role of environmental reservoirs in human campylobacteriosis. Int J Environ Res Public Health.

[2] Ravel A, Davidson VJ, Ruzante JM, Fazil A (2010). Foodborne proportion of gastrointestinal illness: estimates from a Canadian expert elicitation survey. Foodborne Pathog Dis.

[3] Havelaar AH, Galindo AV, Kurowicka D, Cooke RM (2008). Attribution of foodborne pathogens using structured expert elicitation. Foodborne Pathog Dis.

[4] Vally H, Glass K, Ford L, Hall G, Kirk MD, Shadbolt C (2014). Proportion of illness acquired by foodborne transmission for nine enteric pathogens in Australia: an expert elicitation. Foodborne Pathog Dis.

[5] Domingues AR, Pires SM, Halasa T, Hald T (2012). Source attribution of human campylobacteriosis using a meta-analysis of case-control studies of sporadic infections. Epidemiol Infect.

[6] Fajo-Pascual M, Godoy P, Ferrero-Cancer M, Wymore K (2010). Case-control study of risk factors for sporadic Campylobacter infections in northeastern Spain. Eur J Public Health.

[7] Thomas MK, Murray R, Flockhart L, Pintar K, Pollari F, Fazil A (2013). Estimates of the Burden of Foodborne Illness in Canada for 30 Specified Pathogens and Unspecified Agents, Circa 2006. Foodborne Pathog Dis.

[8] Michaud S, Menard S, Arbeit RD (2004). Campylobacteriosis, Eastern Townships, Quebec. Emerg Infect Dis.

[9] Levesque S, Fournier E, Carrier N, Frost E, Arbeit RD, Michaud S (2013). Campylobacteriosis in urban versus rural areas: a case-case study integrated with molecular typing to validate risk factors and to attribute sources of infection. PLoS One.

[10] Galanis E, Mak S, Otterstatter M, Taylor M, Zubel M, Takaro TK (2014). The association between campylobacteriosis, agriculture and drinking water: a case-case study in a region of British Columbia, Canada, 2005–2009. Epidemiol Infect.

[11] Murphy HM, Thomas KM, Schmidt P, Medeiros DT, McFadyen S, Pintar KDM (2015). Estimates of acute gastrointestinal illness due to Giardia, Cryptosporidium, Campylobacter, E. coli O157 and norovirus associated with private wells and small water systems in Canada. Epidemiol Infect.

[12] Gillespie IA, O'Brien SJ, Frost JA, Adak GK, Horby P, Swan AV (2002). A case-case comparison of Campylobacter coli and Campylobacter jejuni infection: a tool for generating hypotheses. Emerg Infect Dis.

[13] Naumova EN, Jagai JS, Matyas B, DeMaria A, MacNeill IB, Griffiths JK (2007). Seasonality in six enterically transmitted diseases and ambient temperature. Epidemiol Infect.

[14] Nylen G, Dunstan F, Palmer SR, Andersson Y, Bager F, Cowden J (2002). The seasonal distribution of campylobacter infection in nine European countries and New Zealand. Epidemiol Infect.

[15] Nelson W, Harris B (2011). Campylobacteriosis rates show age-related static bimodal and seasonality trends. N Z Med J.

[16] White AN, Kinlin LM, Johnson C, Spain CV, Ng V, Fisman DN (2009). Environmental determinants of campylobacteriosis risk in Philadelphia from 1994 to 2007. Ecohealth.

[17] Arsenault J, Michel P, Berke O, Ravel A, Gosselin P (2012). Environmental characteristics associated with campylobacteriosis: accounting for the effect of age and season. Epidemiol Infect.

[18] Doorduyn Y, Van Den Brandhof WE, Van Duynhoven YT, Breukink BJ, Wagenaar JA, Van Pelt W (2010). Risk factors for indigenous Campylobacter jejuni and Campylobacter coli infections in The Netherlands: a case-control study. Epidemiol Infect.

[19] Fullerton KE, Ingram LA, Jones TF, Anderson BJ, McCarthy PV, Hurd S (2007). Sporadic campylobacter infection in infants: a population-based surveillance case-control study. Pediatr Infect Dis J.

[20] MacRitchie LA, Hunter CJ, Strachan NJC (2013). A population-based exposure assessment of risk factors associated with gastrointestinal pathogens: a Campylobacter study. Epidemiol Infect.

[21] Unicomb LE, Dalton CB, Gilbert GL, Becker NG, Patel MS (2008). Age-specific risk factors for sporadic Campylobacter infection in regional Australia. Foodborne Pathog Dis.

[22] Fitzenberger J, Uphoff H, Gawrich S, Hauri AM. Urban-rural differences of age- and species-specific campylobacteriosis incidence, Hesse, Germany, July 2005 - June 2006. Euro Surveill. 2010;15(42). 10.2807/ese.15.42.19693-en 21034721

[23] Potter RC, Kaneene JB, Hall WN (2003). Risk factors for sporadic Campylobacter jejuni infections in rural michigan: a prospective case-control study. Am J Public Health.

[24] Ravel A, Nesbitt A, Marshall B, Sittler N, Pollari F (2011). Description and burden of travel-related cases caused by enteropathogens reported in a Canadian community. J Travel Med.

[25] David JM, Ravel A, Nesbitt A, Pintar K, Pollari F (2014). Assessing multiple foodborne, waterborne and environmental exposures of healthy people to potential enteric pathogen sources: effect of age, gender, season, and recall period. Epidemiol Infect.

[26] Hosmer DW, Lemeshow S (2000). Applied Logistc Regression.

[27] Kuritz SJ, Landis JR (1988). Attributable Risk-Estimation from Matched Case Control Data. Biometrics.

[28] Dupont WD (1988). Power Calculations for Matched Case Control Studies. Biometrics.

[29] Karenlampi R, Rautelin H, Schonberg-Norio D, Paulin L, Hanninen ML (2007). Longitudinal study of Finnish Campylobacter jejuni and C. coli isolates from humans, using multilocus sequence typing, including comparison with epidemiological data and isolates from poultry and cattle. Appl Environ Microbiol.

[30] Mughini Gras L, Smid JH, Wagenaar JA, de Boer AG, Havelaar AH, Friesema IH (2012). Risk factors for campylobacteriosis of chicken, ruminant, and environmental origin: a combined case-control and source attribution analysis. PLoS One.

[31] Breitenmoser A, Fretz R, Schmid J, Besl A, Etter R (2011). Outbreak of acute gastroenteritis due to a washwater-contaminated water supply, Switzerland, 2008. J Water Health.

[32] Karagiannis I, Sideroglou T, Gkolfinopoulou K, Tsouri A, Lampousaki D, Velonakis EN (2010). A waterborne Campylobacter jejuni outbreak on a Greek island. Epidemiol Infect.

[33] O'Reilly CE, Bowen AB, Perez NE, Sarisky JP, Shepherd CA, Miller MD (2007). A waterborne outbreak of gastroenteritis with multiple etiologies among resort island visitors and residents: Ohio, 2004. Clin Infect Dis.

[34] Zeigler M, Claar C, Rice D, Davis J, Frazier T, Turner A (2014). Outbreak of campylobacteriosis associated with a long-distance obstacle adventure race--Nevada, October 2012. MMWR.

[35] Jakopanec I, Borgen K, Vold L, Lund H, Forseth T, Hannula R (2008). A large waterborne outbreak of campylobacteriosis in Norway: The need to focus on distribution system safety. BMC Infect Dis.

[36] Gubbels SM, Kuhn KG, Larsson JT, Adelhardt M, Engberg J, Ingildsen P (2012). A waterborne outbreak with a single clone of Campylobacter jejuni in the Danish town of Koge in May 2010. Scand J Infect Dis.

[37] Denno DM, Keene WE, Hutter CM, Koepsell JK, Patnode M, Flodin-Hursh D (2009). Tri-county comprehensive assessment of risk factors for sporadic reportable bacterial enteric infection in children. J Infect Dis.

[38] Mellou K, Sourtzi P, Tsakris A, Saroglou G, Velonakis E (2010). Risk factors for sporadic Campylobacter jejuni infections in children in a Greek region. Epidemiol Infect.

[39] Butler AJ, Thomas MK, Pintar KDM (2015). Expert Elicitation as a Means to Attribute 28 Enteric Pathogens to Foodborne, Waterborne, Animal Contact, and Person-to-Person Transmission Routes in Canada. Foodborne Pathog Dis.

[40] Carrique-Mas J, Andersson Y, Hjertqvist M, Svensson A, Torner A, Giesecke J (2005). Risk factors for domestic sporadic campylobacteriosis among young children in Sweden. Scand J Infect Dis.

[41] Kapperud G, Espeland G, Wahl E, Walde A, Herikstad H, Gustavsen S (2003). Factors associated with increased and decreased risk of Campylobacter infection: a prospective case-control study in Norway. Am J Epidemiol.

[42] Schonberg-Norio D, Takkinen J, Hanninen ML, Katila ML, Kaukoranta SS, Mattila L (2004). Swimming and Campylobacter infections. Emerg Infect Dis.

[43] Blaser MJ, Smith PF, Wang WLL, Hoff JC (1986). Inactivation of Campylobacter-Jejuni by Chlorine and Monochloramine. Appl Environ Microbiol.

[44] Nygard K, Andersson Y, Rottingen JA, Svensson A, Lindback J, Kistemann T (2004). Association between environmental risk factors and campylobacter infections in Sweden. Epidemiol Infect.

[45] Canada H (2014). Guidelines for Canadian drinking water quality - summary table. Water and Air Quality Bureau HEaCSB.

[46] Godoy P, Artigues A, Nuin C, Aramburu J, Perez M, Dominguez A (2002). Outbreak of gastroenteritis caused by Campylobacter jejuni transmitted through drinking water. Med Clin Barcelona.

[47] Charlett A, Cowden JM, Frost JA, Gillespie IA, Millward J, Neal KR (2002). Ciprofloxacin resistance in Campylobacter jejuni: case-case analysis as a tool for elucidating risks at home and abroad. J Antimicrob Chemoth.

[48] Mughini-Gras L, Smid JH, Wagenaar JA, DE Boer A, Havelaar AH, Friesema IH (2014). Campylobacteriosis in returning travellers and potential secondary transmission of exotic strains. Epidemiol Infect.

[49] Evans MR, Northey G, Sarvotham TS, Hopkins AL, Rigby CJ, Thomas DR (2009). Risk factors for ciprofloxacin-resistant Campylobacter infection in Wales. J Antimicrob Chemoth.

[50] Evans MR, Ribeiro CD, Salmon RL (2003). Hazards of healthy living: bottled water and salad vegetables as risk factors for Campylobacter infection. Emerg Infect Dis.

[51] Warburton DW (1993). A review of the microbiological quality of bottled water sold in Canada. Part 2. The need for more stringent standards and regulations. Can J Microbiol.

[52] Canada PHAo (2009). Canadian Integrated Surveillance Report: Salmonella, Campylobacter, verotoxigenic E. coli and Shigella, from 2000 to 2004. Can Commun Dis Rep.

[53] Taylor EV, Herman KM, Ailes EC, Fitzgerald C, Yoder JS, Mahon BE (2013). Common source outbreaks of Campylobacter infection in the USA, 1997–2008. Epidemiol Infect.

[54] Pires SM, Vigre H, Makela P, Hald T (2010). Using Outbreak Data for Source Attribution of Human Salmonellosis and Campylobacteriosis in Europe. Foodborne Pathog Dis.

[55] Wilson N (2005). A Systematic Review of the Aetiology of Human Campylobacteriosis in New Zealand.

[56] Bessell PR, Matthews L, Smith-Palmer A, Rotariu O, Strachan NJC, Forbes KJ (2010). Geographic determinants of reported human Campylobacter infections in Scotland. BMC Public Health.

[57] Bemis K, Marcus R, Nadler JL (2014). Socioeconomic Status and Campylobacteriosis, Connecticut, USA, 1999–2009. Emerg Infect Dis.

[58] Danis K, Di Renzi M, O'Neill W, Smyth B, McKeown P, Foley B, et al. Risk factors for sporadic Campylobacter infection: an all-Ireland case-control study. Euro Surveill. 2009;14(7). 19232225

[59] Effler P, Ieong MC, Kimura A, Nakata M, Burr R, Cremer E (2001). Sporadic Campylobacter jejuni infections in Hawaii: associations with prior antibiotic use and commercially prepared chicken. J Infect Dis.

[60] Koningstein M, Simonsen J, Helms M, Hald T, Molbak K (2011). Antimicrobial use: a risk factor or a protective factor for acquiring campylobacteriosis?. Clin Infect Dis.

[61] Tam CC, Higgins CD, Neal KR, Rodrigues LC, Millership SE, O'Brien SJ (2009). Chicken consumption and use of acid-suppressing medications as risk factors for Campylobacter enteritis, England. Emerg Infect Dis.

[62] Mossong J, Mughini-Gras L, Penny C, Devaux A, Olinger C, Losch S (2016). Human Campylobacteriosis in Luxembourg, 2010–2013: a case-control study combined with Multilocus sequence typing for source attribution and risk factor analysis. Sci Rep Uk.

[63] Stafford RJ, Schluter P, Kirk M, Wilson A, Unicomb L, Ashbolt R (2007). A multi-centre prospective case-control study of campylobacter infection in persons aged 5 years and older in Australia. Epidemiol Infect.

