# Supplementary material for: Non food-related risk factors of campylobacteriosis in Canada: a matched case-control study
Source: BMC Public Health. 2016 Sep 27;16:1016. doi: 10.1186/s12889-016-3679-4 (PMC5039884; doi:10.1186/s12889-016-3679-4)
Supplement: Additional file 1: — Campylobacter Worksheet. (PDF 45 kb) [file 12889_2016_3679_MOESM1_ESM.pdf]

# Campylobacter Worksheet

**CASE DETAILS**

| | | | | |
|---|---|---|---|---|
| Subtype: | ☐ C. jejuni ☐ C. arcobacter<br>☐ C. coli ☐ C. fetus<br>☐ Non-groupable/typable<br>☐ Other, specify: | | Date Received | yyyy/mm/dd |
| | | | Investigating PHI: | |
| | | | CD #: | |
| DNA Pattern | Phage Type: | PFG Type: | iPHIS Case ID #: | |
| Source: | ☐ Blood ☐ Stool<br>☐ Urine ☐ Other | | Outbreak #: | |

Case Classification

| | | | | | |
|---|---|---|---|---|---|
| ☐ Suspect | Date: yyyy/mm/dd | ☐ Confirmed | Date: yyyy/mm/dd | ☐ Carrier | Date: yyyy/mm/dd |

## Client Demographics ☐ *Case* ☐ *Contact* ☐ *Both*

**DEMOGRAPHICS**

| | | | | | |
|---|---|---|---|---|---|
| Name | *first* | *last* | | DOB: | *yyyy/mm/dd* |
| Health Card #: | | Gender: | ☐ Male<br>☐ Female<br>☐ Transgender | Age: | |
| Address Type: | ☐ Home ☐ Other: | | | Marital Status: | |
| Address: | | | | | |
| City: | | Province: | | Postal Code: | |

*Telecommunication*

| | | | | | |
|---|---|---|---|---|---|
| Home: | | Business: | | Other: | |

| *Employment* | 1 | 2 | 3 |
|---|---|---|---|
| Occupation: | | | |
| Employer/School: | | | |
| Address: | | | |
| Telephone: | | | |
| Estimated Start Date: | *yyyy/mm/dd* | *yyyy/mm/dd* | *yyyy/mm/dd* |

**CASE DETAILS**

| | | | |
|---|---|---|---|
| Attending MD: | | Telephone: | |
| Family MD: | | Telephone: | |

*Hospitalization* ☐ Yes ☐ No ☐ Unsure Specify:

| | | | |
|---|---|---|---|
| Admission Date: | *yyyy/mm/dd* | Discharged Date: | *yyyy/mm/dd* |

**OUT-COM**

| | | | |
|---|---|---|---|
| *Date of Death:* | *yyyy/mm/dd* | Cause of Death: | |

## Case Management *Onset Date:* *Recovery Date:*

**SYMPTOMS**

*Symptoms:* *yyyy/mm/dd (24h00)*

| | | | | | | |
|---|---|---|---|---|---|---|
| Asymptomatic: (NO symptoms reported) | ☐ Yes ☐ No ☐ Unsure | Onset Date: Time: | | Duration: | 0 | ☒ days ☐ hours |
| Abdominal Pain: | ☐Yes ☐ No ☐ Unsure | Onset Date: Time: | | Duration: | | ☐ days ☐ hours |
| Diarrhea: | ☐Yes ☐ No ☐ Unsure | Onset Date: Time: | | Duration: | | ☐ days ☐ hours |
| Diarrhea–bloody: | ☐Yes ☐ No ☐ Unsure | Onset Date: Time: | | Duration: | | ☐ days ☐ hours |
| Fever: | ☐Yes ☐ No ☐ Unsure | Onset Date: Time: | | Duration: | | ☐ days ☐ hours |

# Campylobacter Worksheet

| | | | | | | |
|---|---|---|---|---|---|---|
| Headache: | ☐Yes ☐ No ☐ Unsure | Onset Date:<br>Time: | | Duration: | | ☐ days ☐ hours |
| Malaise: | ☐Yes ☐ No ☐ Unsure | Onset Date:<br>Time: | | Duration: | | ☐ days ☐ hours |
| Nausea: | ☐Yes ☐ No ☐ Unsure | Onset Date:<br>Time: | | Duration: | | ☐ days ☐ hours |
| Vomiting: | ☐Yes ☐ No ☐ Unsure | Onset Date:<br>Time: | | Duration: | | ☐ days ☐ hours |
| Other: | ☐Yes ☐ No ☐ Unsure | Onset Date:<br>Time: | | Duration: | | ☐ days ☐ hours |
| Other: | ☐Yes ☐ No ☐ Unsure | Onset Date:<br>Time: | | Duration: | | ☐ days ☐ hours |
| Other: | ☐Yes ☐ No ☐ Unsure | Onset Date:<br>Time: | | Duration: | | ☐ days ☐ hours |
| Other: | ☐Yes ☐ No ☐ Unsure | Onset Date:<br>Time: | | Duration: | | ☐ days ☐ hours |

*Household Contacts:* Family members ill? ☐Yes ☐ No ☐ Unsure ☐ Lives alone

| *Person:* | *Age* | *Gender* | *Symptoms: (onset date:)* |
|---|---|---|---|
| | | | |

| Risk Factors INCUBATION PERIOD: 1-10 days (average 2-5 days) | | | |
|---|---|---|---|
| *These next few questions are about the time frame just before you become ill…* | | | |
| 1. | In the 10 days before illness, which of the following was a source of drinking water for you? Please indicate the main source. | | |
| | *Main* | *Other* | ☐ *Did not know* |
| | ☐ | ☐ | Private well----*Type of well:* ☐ *Dug* ☐ *Drilled (deep)* ☐ *Drilled (shallow)* ☐ *Other:* |
| | ☐ | ☐ | Municipal/ City Water |
| | ☐ | ☐ | Bottled Water |
| | ☐ | ☐ | Other water source: |
| 2. | Do you use an in-home treatment system for your drinking water | | ☐Yes ☐ No ☐ Unsure |
| | *If yes*, is it: ☐ Reverse Osmosis ☐ Ultraviolet Light ☐ On-tap Filter ☐ Water Pitcher Filter (such as Brita) ☐ Other: | | |
| 3. | In the 10 days before illness, did you drink untreated/raw water (other than your home)? | | ☐Yes ☐ No ☐ Unsure |
| | *If yes*, where? | | |

# Campylobacter Worksheet

| | | |
|---|---|---|
| 4. | In the 10 days before illness, did you swim in/go into any of the following: the ocean, a lake, a river, a pool, or a hot tub? | ☐Yes ☐ No ☐ Unsure |
| | *If yes,* where? ☐ Ocean ☐ Lake ☐ River ☐ Pool ☐ Hot Tub ☐ Other: | |
| 5. | In the 10 days before illness, did you go canoeing, kayaking, hiking or camping? | ☐Yes ☐ No ☐ Unsure |
| | *If yes,* where? | |
| 6. | In the 10 days before illness, did you drink or eat any unpasteurized milk, juice or dairy products? | ☐Yes ☐ No ☐ Unsure |
| | *If yes,* what? ☐ Milk ☐ Juice ☐ Dairy Products ☐ Other: | |
| 7. | In the 10 days before illness, did you eat meat from any place other than the grocery store?(ie. Hunting, butcher shop, private kill) | ☐Yes ☐ No ☐ Unsure |
| | *If yes,* where? ☐ Hunting ☐ Butcher shop ☐ Private kill ☐ Other: | |
| 8. | Where did you shop for your food eaten during the week before your illness? | |

| | *Main* | *Other* | *Location of purchase:* | |
|---|---|---|---|---|
| | ☐ | ☐ | Supermarket: | Date: |
| | ☐ | ☐ | Farmers Market: | Date: |
| | ☐ | ☐ | Butcher Shop:: | Date: |
| | ☐ | ☐ | Farm (laneway): | Date: |
| | ☐ | ☐ | Other: | Date: |

| | | |
|---|---|---|
| 9. | In the 10 days before illness, did you have or attend a barbeque? | ☐Yes ☐ No ☐ Unsure |
| | *If yes,* where? Date: | |
| 10. | In the 10 days before illness, did you attend any social gatherings (wedding, receptions, showers, parties, festivals, fairs, etc)? | ☐Yes ☐ No ☐ Unsure |
| | *If yes,* what/where? Date: | |
| 11. | In the 10 days before illness, did you live on a farm or country property? | ☐Yes ☐ No ☐ Unsure |
| | *If yes,* where?<br>*Type of animals in contact with:* | |
| 12. | In the 10 days before illness, did you visit a farm (other than your own), petting zoo or fair? | ☐Yes ☐ No ☐ Unsure |
| | *If yes,* where?<br>*Type of animals in contact with:* | |
| 13. | In the 10 days before illness, did you garden? | ☐Yes ☐ No ☐ Unsure |
| | *If yes,* date: | |

# Campylobacter Worksheet

| | | | | |
|---|---|---|---|---|
| 14. | In the 10 days before illness, did you have any contact with household pets (including reptiles and hedgehogs)? | | | ☐Yes ☐ No ☐ Unsure |
| | *If yes*, type of animal: | *Was the animal ill?*: | *Did you have contact with its feces?* | |
| | ☐ Bird | ☐Yes ☐ No ☐ Unsure | ☐Yes ☐ No ☐ Unsure | |
| | ☐ Cat | ☐Yes ☐ No ☐ Unsure | ☐Yes ☐ No ☐ Unsure | |
| | ☐ Dog | ☐Yes ☐ No ☐ Unsure | ☐Yes ☐ No ☐ Unsure | |
| | ☐ Reptile | ☐Yes ☐ No ☐ Unsure | ☐Yes ☐ No ☐ Unsure | |
| | ☐ Rodent | ☐Yes ☐ No ☐ Unsure | ☐Yes ☐ No ☐ Unsure | |
| | ☐ Other, specify: | ☐Yes ☐ No ☐ Unsure | ☐Yes ☐ No ☐ Unsure | |
| 15. | In the 10 days before illness, did you travel? | | | ☐Yes ☐ No ☐ Unsure |
| | *If yes*, where: Dates: to | | | |
| | *Type of travel:* *☐ Cruise* *☐ Airline, specify:* *Flight No.* <br> *☐ Train* *☐ Bus* *☐ Car* *☐ Other:* | | | |
| | *Did you stay at a resort?* | | | ☐Yes ☐ No ☐ Unsure |
| | *If yes*, name of resort: | | | |
| 16. | In the 10 days before illness, did you know anyone else with a diarrheal illness?(other than household contacts) | | | ☐Yes ☐ No ☐ Unsure |
| | *If yes*, who: Date: | | | |
| 17. | In the 10 days before illness, did you eat any food prepared outside of the home, for example? *(check all that apply)* <br> ☐ Did not eat food prepared outside of the home. | | | |
| | ☐ Eat in Restaurant | Name: | Address: | Date: |
| | ☐ Eat in Cafeteria | Name: | Address: | Date: |
| | ☐ Deli | Name: | Address: | Date: |
| | ☐ Ready-to-eat | Name: | Address: | Date: |
| | ☐ Fast Food | Name: | Address: | Date: |
| | ☐ Food Vendor <br> ie. Markets, special event, hot dog cart | Name: | Address: | Date: |

# Campylobacter Worksheet

*Food History:*
*18. These next few questions are about the time frame just before you become ill…*

DAY 1:_______/___/___
yyyy/mm/dd

| Time of Meal | Meal | Ate at home | Ate outside of home | Outside location | Foods eaten |
|---|---|---|---|---|---|
| ___________ | Breakfast | ☐ | ☐ | ________________ | ____________________ |
| ___________ | Lunch | ☐ | ☐ | ________________ | ____________________ |
| ___________ | Dinner | ☐ | ☐ | ________________ | ____________________ |
| ___________ | Other | ☐ | ☐ | ________________ | ____________________ |

DAY 2:_______/___/___
yyyy/mm/dd

| Time of Meal | Meal | Ate at home | Ate outside of home | Outside location | Foods eaten |
|---|---|---|---|---|---|
| ___________ | Breakfast | ☐ | ☐ | ________________ | ____________________ |
| ___________ | Lunch | ☐ | ☐ | ________________ | ____________________ |
| ___________ | Dinner | ☐ | ☐ | ________________ | ____________________ |
| _____________ | Other | ☐ | ☐ | ___________________ | ____________________ |

DAY 3:_______/___/___
yyyy/mm/dd

| Time of Meal | Meal | Ate at home | Ate outside of home | Outside location | Foods eaten |
|---|---|---|---|---|---|
| ___________ | Breakfast | ☐ | ☐ | ________________ | ____________________ |
| ___________ | Lunch | ☐ | ☐ | ________________ | ____________________ |
| ___________ | Dinner | ☐ | ☐ | ________________ | ____________________ |
| ___________ | Other | ☐ | ☐ | ________________ | ____________________ |

DAY 4:_______/___/___
yyyy/mm/dd

| Time of Meal | Meal | Ate at home | Ate outside of home | Outside location | Foods eaten |
|---|---|---|---|---|---|
| ___________ | Breakfast | ☐ | ☐ | ________________ | ____________________ |
| ___________ | Lunch | ☐ | ☐ | ________________ | ____________________ |
| ___________ | Dinner | ☐ | ☐ | ________________ | ____________________ |
| ___________ | Other | ☐ | ☐ | ________________ | ____________________ |

| | | |
|---|---|---|
| 19. | In the 10 days before illness, do you recall eating any food that was undercooked? | ☐Yes ☐ No ☐ Unsure |
| | *If yes*, what: *Date:* | |
| 20.. | In the 10 days before illness, do you recall eating any food that did not taste right (spoiled)? | ☐Yes ☐ No ☐ Unsure |
| | *If yes*, what: *Date:* | |
| 21. | PHI: Most likely source of infection: | |

Interventions (consultations, education, exclusion, food recall, etc.):

| *Date* | *Type* | *Intervention Notes:* |
|---|---|---|
| | | |
| | | |
| | | |
| | | |
| | | |
| | | |

| | |
|---|---|
| Interview Completion Date: | |

**Notes:**

| | |
|---|---|
| Date Case Closed: | |
